# The Utility of ICD-11 and DSM-5 Traits for Differentiating Patients With Personality Disorders From Other Clinical Groups

**DOI:** 10.3389/fpsyt.2021.633882

**Published:** 2021-04-16

**Authors:** Rute Pires, Joana Henriques-Calado, Ana Sousa Ferreira, Bo Bach, Marco Paulino, João Gama Marques, Ana Ribeiro Moreira, Jaime Grácio, Bruno Gonçalves

**Affiliations:** ^1^Faculdade de Psicologia, Universidade de Lisboa, Lisbon, Portugal; ^2^CICPSI, Faculdade de Psicologia, Universidade de Lisboa, Lisbon, Portugal; ^3^Instituto Universitário de Lisboa - Business Research Unit (BRU-IUL), Lisbon, Portugal; ^4^Center for Personality Disorder Research, Psychiatric Research Unit, Slagelse, Denmark; ^5^Faculdade de Medicina, Universidade de Lisboa, Lisbon, Portugal; ^6^Clínica de Psiquiatria Geral e Transcultural, Hospital Júlio de Matos, Centro Hospitalar Psiquiátrico de Lisboa, Lisbon, Portugal; ^7^Clínica Universitária de Psiquiatra e Psicologia Médica, Faculdade de Medicina, Universidade de Lisboa, Lisbon, Portugal; ^8^Centro Hospitalar de Lisboa Ocidental, Hospital de Egas Moniz, Lisbon, Portugal; ^9^Champalimaud Clinical Centre, Champalimaud Centre for the Unknown, Champalimaud Foundation, Lisbon, Portugal; ^10^Champalimaud Research, Champalimaud Centre for the Unknown, Champalimaud Foundation, Lisbon, Portugal; ^11^NOVA Medical School/Faculdade de Ciências Médicas, Universidade Nova de Lisboa, Lisbon, Portugal

**Keywords:** ICD-11 classification of personality disorders, DSM-5 alternative model for personality disorders, personality disorders, severity, personality traits, PID5BF+M

## Abstract

The ICD-11 Classification of Personality Disorders delineates five trait domain qualifiers (i.e., negative affectivity, detachment, dissociality, disinhibition, and anankastia), whereas the DSM-5 Alternative Model of Personality Disorders also delineates a separate domain of psychoticism. These six combined traits not only characterize individual stylistic features, but also the severity of their maladaptive expressions. It was, therefore, the aim of this study to investigate the utility of ICD-11 and DSM-5 trait domains to differentiate patients with personality disorders (PD) from patients with other mental disorders (non-PD). The Personality Inventory for DSM-5 Brief Form Plus (PID5BF+M) was administered to a sample of patients diagnosed with a personality disorder (*N* = 124, *M*_age_ = 42.21, 42.7% females) along with a sample of patients diagnosed with other mental disorders (*N* = 335, *M*_age_ = 44.83, 46.6% females). Group differences were explored using the independent sample *t* test or the Mann–Whitney *U* test for independent samples, and discriminant factor analysis was used to maximize group differences for each trait domain and facet score. The PD group showed significantly higher scores for the total PID5BF+M composite score, for the trait domains of negative affectivity, antagonism/dissociality, and disinhibition and for the trait facets of emotional lability, manipulativeness, deceitfulness, and impulsivity. The trait domains of disinhibition, negative affectivity, and antagonism/dissociality as well as the trait facets of impulsivity, deceitfulness, emotional lability, and manipulativeness were the best discriminators between PD and non-PD patients. The global PID5BF+M composite score was also one of the best discriminators supporting its potential as a global severity index for detecting personality dysfunction. Finally, high scores in three or more of the 18 PID5BF+M facets suggested the possible presence of a PD diagnosis. Despite some limitations, our findings suggest that the ICD-11 and DSM-5 traits have the potential to specifically describe the stylistic features that characterize individuals with PD, including the severity of their maladaptive expressions.

## Introduction

There is broad consensus within the scientific community as to the supremacy of dimensional classification models over categorical models in the diagnosis of personality disorders (PD) (1, 2). Moreover, a growing body of research suggests that the global severity of personality dysfunction should be central for PD diagnosis (3–5) while individual maladaptive expressions are best described in terms of specific traits (6, 7). A PD classification system based on severity is expected to simplify the diagnostic process and is far more beneficial to clinical practice, allowing for a clear identification of those who are more disturbed and require a more intensive intervention (e.g., hospitalization vs. outpatient treatment). However, the stylistic manifestations of personality dysfunction may reveal specific areas of difficulty and are, therefore, also important to identify. Style indicates the likely expression of the pathology and gears the clinician toward the most appropriate type of intervention (3, 6, 8).

Reflecting this trend, the recently released ICD-11 Classification of Personality Disorders (9) and the DSM-5 Alternative Model of Personality Disorders (AMPD) (10) consider impairments in self and interpersonal functioning as the core feature of PD and delineate levels of dysfunction. As mentioned, personality dysfunction may have different phenotypic manifestations with implications for treatment and outcomes. Therefore, both classification systems use pathological traits to characterize the stylistic expression of personality dysfunction. In the ICD-11 model, the traits are specifiers of personality dysfunction (i.e., severe personality dysfunction is expected to be associated with several pathological traits) while the DSM-5 model defines constellations of traits that characterize six personality disorders (e.g., for the diagnosis of borderline personality disorder, in addition to moderate or greater personality dysfunction, four or more of seven pathological traits must be present and at least one must be impulsivity, risk taking, or hostility) (9, 10). The trait domains considered in both models are negative affectivity, detachment, antagonism/dissociality, and disinhibition. The two models particularly differ in terms of the psychoticism domain, which is not considered a personality trait domain in the ICD-11, and in terms of the anankastia domain, whose equivalent in the DSM-5 (compulsivity) was not retained in the final AMPD model for reasons of parsimony (9, 10). Additionally, the DSM-5 recognizes facets within each trait domain, and the ICD-11 does not. Both models have made efforts to operationalize these specific trait features. The Personality Inventory for ICD-11 (11) and the Personality Inventory for DSM-5 (PID5) (12) have proven to efficiently assess the maladaptive traits of each model, helping clinicians to easily capture the most salient traits in each patient.

Considering the similarity of both classification systems, it would be helpful to clinicians if these two systems were harmonized (13, 14). In fact, as previously mentioned, the preliminary versions of the DSM-5 included a compulsivity domain, akin to anankastia (15). Currently, the DSM-5 addresses compulsivity/anankastia in terms of a low score in the disinhibition domain (i.e., rigid perfectionism) and a high score in perseveration (10). However, experts stress that rigid perfectionism and perseveration do not capture the complexity of the compulsivity dimension (16, 17), and research findings support a distinct compulsivity/anankastia domain (13, 14, 18). In contrast to the DSM-5, the ICD-11 does not consider psychoticism as a personality feature as it describes mental functioning (bizarre behavior, unusual thoughts, and experiences) that characterizes schizophrenic spectrum disorders. Nonetheless, the DSM-5 psychoticism domain captures features of schizotypy that are close to normal functioning (i.e., unconventionality in appearance and thinking), therefore ranging from atypical normal functioning to more extreme schizophrenic-like features.

Recently, Kerber et al. (19) developed the Personality Inventory for the DSM-5—Brief Form Plus (PID5BF+), an algorithm that assesses the DSM-5 and ICD-11 six trait domains (negative affectivity, detachment, antagonism/dissociality, disinhibition, anankastia, and psychoticism) and 17 facets. In the PID5BF+, the anankastia domain aligns with the DSM-5 algorithm and consists of the rigid perfectionism and perseveration facets. To better capture the features of the ICD-11 domain of anankastia, Bach et al. (14) developed a modified version of the PID5BF+, the PID5BF+M, in which the perseveration facet, which contributes to the negative affectivity domain, was excluded and the orderliness, rigidity, and perfectionism sub-facets of the original rigid perfectionism facet were added. These three new anankastia facets are in keeping with the initial 37-facet version of the DSM-5 trait model that included the compulsivity domain (15). Apart from these changes in the composition of the anankastia domain, the other PID5BF+ domains have remained unchanged. Therefore, each PID5BF+M domain is composed of three facets, and each facet is composed of two items. The PID5BF+M was validated with international PID5 data. Although the ICD-11 classification system does not describe personality at a facet level, research with the PID5BF+M (14) reveals that the ICD-11 trait domain may be adequately characterized by means of DSM-5 trait facets.

According to the ICD-11 approach, global PD severity rather than specific trait qualifiers are meant to differentiate PD from non-PD patients. Nevertheless, it is well-established that severity represents the global quality (“g-factor”) that links all maladaptive personality features (3, 6, 8, 20). Moreover, the ICD-11 PD classification states that individuals with more severe personality disturbance tend to have a greater number of prominent trait domains (9). This implies higher scores across the trait domains, thus indicating more complexity, which, in turn, indicates PD severity. Therefore, the number, complexity, and severity of maladaptive traits indicate the global severity of personality dysfunction (3). This is also consistent with the official PID5 user's guide, which literally instructs users to calculate the overall personality dysfunction score (i.e., total and individual PID5 scores) to track changes in the severity of the individual's personality dysfunction over time (10). Moreover, research supports the understanding that PID-5 traits are substantially related to measures of functional impairment (21).

The current study sought to investigate the utility of the PID5BF+M total score along with specific domain and facet scores in differentiating patients with PDs from other psychiatric patients. The use of the PID5BF+ as a proxy of severity is particularly supported in a comparative study by Zimmermann et al. (22), which found the total PID5BF+ score to align well with a number of PD severity measures.

## Materials and Methods

### Participants and Procedures

The sample of patients with PD consisted of 124 patients aged between 18 and 68 years (*M*_age_ = 42.21 years, SD = 11.76 years, 57.3% males, 42.7% females), 64.5% of whom had a cluster B diagnosis (56.6% had borderline personality disorder). In the PD sample, 56.5% of the patients had comorbid psychopathology. The most common comorbid disorders included substance-related and addictive (22.6%), depressive (17.7%), and bipolar disorders (10.5%).

The sample of patients with other diagnoses (39.1% depressive, 29.6% substance-related and addictive, and 15.5% bipolar and related disorders) was composed of 335 patients, aged 18 to 76 years (*M*_age_ = 44.83 years, SD = 12.59 years, 53.4% males, 46.6% females). Individuals with a comorbid PD were excluded from this sample.

Data collection was carried out within the scope of the adaptation of the PID5 (12) for Portugal. The study design was approved by the ethics committees of the affiliated and host institutions, and the research protocol consisted of a sociodemographic questionnaire and four personality tests, one of which was the PID5. At the time of data collection, the patients were having treatment at mental health units. In each affiliated mental health institution, a psychiatrist or psychologist (co-authors of this paper) coordinated the sampling procedures. Patients were selected according to their DSM-5 diagnosis and the study's inclusion and exclusion criteria. The study inclusion criteria were adults above 18 years undergoing treatment at mental health units. Diagnoses of intellectual disability, schizophrenia, and major and mild neurocognitive disorders were the exclusion criteria. Some of the patients responded to the research protocol during brief hospitalization periods (for conditions such as eating or affective disorders). Others were outpatients, admitted sequentially in the sample whenever they had a follow-up consultation. Patients were informed that participation in the study was voluntary, that they could withdraw their participation at any time, that no identifying information would be asked, and that the data would be used exclusively in a scientific study.

### Instruments

PID5BF+M: The Modified Version of the PID5BF+ (14). The PID5BF+ (19) is a 34-item self-report that was developed to combine DSM-5 and ICD-11 traits within six domains (negative affectivity, detachment, antagonism/dissociality, disinhibition, anankastia, and psychoticism). The selection of items from the original PID5 item pool was carried out by means of ant colony optimization algorithms [see details in Kerber et al. (19)]. In the PID5BF+M (14), the anankastia domain was revised, and the changes in its operationalization were empirically validated [see details in Bach et al. (14)]. The PID5BF+M comprises 36 items that delineate 18 facets in the six trait domains (three facets per domain). High scores indicate greater dysfunction in a specific trait facet or domain (14). Following the procedure of Zimmermann et al. (22) for the PID5BF+, a total PID5BF+M score was also computed as a global index of personality dysfunction. Our PID5BF+M data was derived from complete PID5 data.

### Data Analysis

Analyses were undertaken with *IBM SPSS Statistics* (v.25, SPSS Inc., Chicago, IL). Descriptive statistics for the facets, domains, and total PID5BF+M score were obtained, and the domains' and total PID5BF+M score reliability was examined through Cronbach's alphas in both PD and non-PD samples. To explore the normality of the scales' distributions, the following criteria were used: skewness, kurtosis, Kolmogorov–Smirnov goodness-of-fit test (*N* > 30), steam and leaf diagrams, and Q-Q plots. Group differences were explored using the independent sample *t* test whenever the PID5BF+M scales followed a normal distribution. Effect sizes were tested through Cohen's *d*, in which the effect size was considered small when *d* ≤ 2.0, medium when 0.20 < *d* ≤ 0.50, large when 0.50 < *d* ≤ 1.0, and very large when *d* > 1.0. The Mann–Whitney *U* test for independent samples was used when the PID5BF+M scales did not follow a normal distribution. Effect sizes were tested through *r* = *Z/*$\sqrt{N}$*, N* = *n*_*PD*_ + *n*_*non*−*PD*_, in which the effect size was considered small when 0.10 ≤ *r* < 0.30, medium when: 0.30 ≤ *r* < 0.50, and large when: *r* ≥ 0.50. Discriminant factor analysis was used to maximize group differences in each PID5BF+M trait domain and facet. Finally, the minimum number of facets with high rates [>2 (23)] that differentiate PD from non-PD diagnosis was examined.

## Results

Cronbach's alphas for the six PID5BF+M domains were moderate although slightly higher in the non-PD sample. In the PD sample, the mean Cronbach's alpha was 0.70, ranging from 0.65 at the lowest level for detachment to 0.76 for psychoticism. Regarding the non-PD sample, the mean Cronbach's alpha was 0.72, ranging from 0.66 at the lowest level for negative affectivity to 0.77 for psychoticism. As for the total PID5BF+M score, in the PD sample the alpha was 0.86, and in the non-PD sample the alpha was 0.89.

Table 1 presents the descriptive statistics, *t* tests, and effect sizes for all the PID5BF+M scales although seven scales (anxiety, withdrawal, intimacy avoidance, manipulativeness, unusual beliefs and experiences, eccentricity, and orderliness) did not lean toward normality. Considering the robustness of parametric statistics against non-normality variables and for the sake of clarity in the results' presentation, the option was taken to present the *t* test results, discussing eventual discrepancies with the Mann–Whitney *U* results whenever necessary.

**Table 1:** PID5BF+M scales' means (*M*), standard deviations (*SD*), *t* tests (*t*) and effect sizes *(d)* in the personality disorder (PD) and non-personality disorder (non-PD) samples.

	**PD** **(*N* = 124)**		**Non-PD** **(*N* = 335)**				
	***M***	***SD***	***M***	***SD***	***t***	***p***	***d***
Emotional lability	1.85	0.87	1.65	0.85	2.26	0.012	0.23
Anxiety	1.98	0.87	1.92	0.88	0.74	0.231	
Separation insecurity	1.53	0.89	1.38	0.87	1.62	0.054	
Withdrawal	1.14	0.83	1.13	0.84	0.15	0.443	
Anhedonia	1.30	0.86	1.18	0.83	1.32	0.100	
Intimacy avoidance	0.88	0.86	1.02	0.88	−1.56	0.060	
Manipulativeness	0.75	0.83	0.58	0.77	2.07	0.020	0.21
Deceitfulness	1.26	0.87	1.06	0.78	2.30	0.011	0.25
Grandiosity	0.88	0.82	0.86	0.81	0.28	0.389	
Irresponsibility	0.83	0.80	0.73	0.78	1.21	0.114	
Impulsivity	1.69	0.81	1.42	0.80	3.20	0.000	0.34
Distractibility	1.71	0.79	1.60	0.76	1.41	0.079	
Unusual beliefs & experiences	1.21	0.87	1.12	0.87	1.01	0.156	
Eccentricity	1.15	0.90	1.03	0.84	1.33	0.093	
Perceptual dysregulation	0.80	0.86	0.75	0.77	0.64	0.261	
Perfectionism	1.50	0.85	1.48	0.85	0.09	0.463	
Rigidity	1.91	0.71	1.83	0.75	1.09	0.138	
Orderliness	1.05	0.76	1.06	0.83	−0.12	0.455	
Negative affectivity	1.79	0.63	1.65	0.60	2.19	0.015	0.23
Detachment	1.11	0.60	1.11	0.64	−0.078	0.469	
Antagonism	0.96	0.64	0.83	0.62	2.05	0.021	0.21
Disinhibition	1.41	0.60	1.25	0.58	2.62	0.005	0.26
Psychoticism	1.05	0.69	0.96	0.68	1.28	0.101	
Anankastia	1.48	0.59	1.46	0.65	0.435	0.332	
Total PID5BF+M	1.30	0.40	1.21	0.43	2.03	0.022	0.21

*Small effect: d ≤ 2.0, medium effect: 0.20 < d ≤ 0.50, large effect: 0.50 < d ≤ 1.0, and very large effect: d > 1.0*.

The PD group showed significantly higher scores for the PID5BF+M total score, the trait domains of negative affectivity, antagonism, and disinhibition, and for the trait facets of emotional lability, manipulativeness, deceitfulness and impulsivity. A small effect size was found for manipulativeness with the Mann–Whitney *U* (*Z* = −2.189, *p* = 0.015, *r* = 0.10), whereas effect sizes tested through Cohen's *d* were medium for the total score and all the trait facets and domains.

Discriminant factor analysis results for the PID5BF+M domains and total are displayed in Table 2. Table 3 shows the 10 more discriminative PID5BF+M traits (PID5BF+M facets and total).

**Table 2:** Discriminant factor analysis for the PID5BF+M trait domains and total in the personality disorder (PD) and non-personality disorder (non-PD) samples.

**Rank**	**Most discriminative variable**	**Mean PD** **(*N* = 124)**	**Mean Non-PD** **(*N* = 335)**	**Percentage of well classified^b^**
1	4 Disinhibition	1.41	1.25	52.5%
2	1 Negative affectivity	1.79	1.65	54.9%
3	3 Antagonism^a^	0.96	0.83	57.1%
4	7 Total PID5BF+M	1.30	1.21	54.0%
5	5 Psychoticism	1.05	.96	52.1%
6	6 Anankastia	1.48	1.46	50.3%
7	2 Detachment	1.11	1.11	50.1%

a *Best predictive capacity*.

b *Two-fold cross-validation*.

**Table 3:** Discriminant factor analysis for the PID5BF+M facets and total in the personality disorder (PD) and non-personality disorder (non-PD) samples.

**Rank**	**Most discriminative variable**	**Mean PD** **(*N* = 124)**	**Mean non-PD** **(*N* = 335)**	**Percentage of well-classified^b^**
1	11 Impulsivity	1.69	1.42	49.0%
2	8 Deceitfulness	1.26	1.06	57.5%
3	1 Emotional lability	1.85	1.65	52.5%
4	7 Manipulativeness^a^	0.75	0.58	60.8%
5	19 Total PID5BF+M	1.30	1.21	54.0%
6	3 Separation insecurity	1.53	1.38	50.1%
7	6 Intimacy avoidance	0.88	1.02	54.2%
8	12 Distractibility	1.71	1.60	55.1%
9	14 Eccentricity	1.15	1.03	56.6%
10	5 Anhedonia	1.30	1.18	50.1%

a *Best predictive capacity*.

b *Twofold cross-validation*.

The trait domains of disinhibition, negative affectivity, and antagonism were the best discriminators among patients with PD and patients with other diagnoses. The antagonism domain (i.e., ICD-11 dissociality) showed the best predictive capacity (57.1%). The PID5BF+M total score discriminated better than the remaining trait domains among PD and non-PD patients.

The trait facets of impulsivity, deceitfulness, emotional lability, manipulativeness, separation insecurity, intimacy avoidance, distractibility, eccentricity, and anhedonia were the best discriminators among patients with PD vs. patients with other diagnoses. Of these, the manipulativeness facet showed the best predictive capacity (60.8%). The PID5BF+M total was the fifth best discriminator among the two groups.

Table 4 presents the number and percentage of individuals with a PD diagnosis or other diagnosis scoring above 2 in the 18 PID5BF+M facets and in the seven traits that significantly differentiated the groups (i.e., negative affectivity, antagonism, disinhibition, emotional lability, manipulativeness, deceitfulness, and impulsivity).

**Table 4:** Number and percentage of individuals from the personality disorder (PD) and non-personality disorder (non-PD) samples with scores above 2 in the 18 PID5BF+M facets and in the seven PID5BF+M facets that differentiate the groups.

			**18 PID5BF+M facets**							
			**>2 in 0**	**>2 in 1**	**>2 in 2**	**>2 in 3**	**Total**			
			**traits**	**traits**	**traits**	**or more traits**				
PD		Count	21	19	18	66	124			
		% within PD	16.9%	15.3%	**14.5%**	**53.2%**	100.0%			
Other diagnosis		Count	87	74	48	123	332			
		% within PD	**26.2%**	**22.3%**	**14.5%**	37.0%	100.0%			
Total		Count	108	93	66	189	456			
		% within PD	23.7%	20.4%	14.5%	41.4%	100.0%			
		**7 PID5BF+M facets**								
		**>2 in 0**	**>2 in 1**	**>2 in 2**	**>2 in 3**	**>2 in 4**	**>2 in 5**	**>2 in 6**	**>2 in 7**	**Total**
		**traits**	**traits**	**traits**	**or more traits**	**or more traits**	**or more traits**	**or more traits**	**or more traits**	
PD	Count	47	25	26	18	7	1	0	0	124
	% within PD	37.9%	20.2%	**21.0%**	**14.5%**	**5.6%**	**0.8%**	0.0%	0.0%	100.0%
Other diagnosis	Count	184	72	51	19	4	2	1	1	334
	% within PD	**55.1%**	**21.6%**	15.3%	5.7%	1.2%	0.6%	0.3%	0.3%	100.0%
Total	Count	231	97	77	37	11	3	1	1	458
	% within PD	50.4%	21.2%	16.8%	8.1%	2.4%	0.7%	0.2%	0.2%	100.0%

*Bold values highlight the number of traits that distinguish the PD group from the non-PD group and above which a PD diagnosis is suggested*.

Elevated scores in three or more of the 18 PID5BF+M facets differentiate the PD group from the other diagnoses group (χ^2^ = 11.124, *p* = 0.011). Considering the seven facets of the PID5BF+M that differentiate the group with PD from the other group, it is sufficient to obtain high results in two or more traits to be assigned to the PD group (χ^2^ = 24.098, *p* = 0.001).

## Discussion

The current study sought to investigate the utility of the PID5BF+M in differentiating patients with PD from other psychiatric patients. A dimensional approach to identifying PD based on severity is proposed by several authors (3, 24, 25), and PD severity is the real differentiator between PD and non-PD in the ICD-11 (9). Although the PID5BF+M addresses trait qualifiers and not the level of PD severity in itself, Zimmermann et al. (22) find that the PID5BF+, from which the PID5BF+M derives, can be used for assessing the severity of PD. This finding is in line with the ICD-11 conceptualization that the number, complexity, and pervasiveness of pathological traits may be an indicator of severity (26) and also with the notion that the AMPD trait model may be used as a measure of severity by summing the number of pathological traits presented (27). Moreover, there is evidence to support the view that severity represents the global quality (g-factor) linking all the maladaptive personality features (6, 8, 20, 21). Therefore, it seems reasonable to predict that maladaptive traits should be more prominent in those with more severe personality dysfunction (i.e., PD). Thus, in this study, the PID5BF+M total score along with the specific domain and facet scores were expected to differentiate PD patients from other psychiatric patients.

The internal consistency of the PID5BF+M six domains and of the total PID5BF+M score in both samples was addressed. Mean Cronbach's alphas ranging from 0.70 in the PD sample to 0.72 in the other mental disorders sample were obtained. The small number of items per domain (each domain is composed of three facets, each with two items) may have accounted for these moderate alphas and supported the decision of not calculating the facets' alphas. However, the total PID5BF+M score showed high internal consistency in both samples, 0.86 in the PD sample and 0.89 in the non-PD sample, thus revealing the reliability of this score in addressing personality dysfunction.

Regarding the samples' descriptives and group differences, all the scales except intimacy avoidance revealed higher means in the PD sample. The PD sample showed significantly higher scores for the total PID5BF+M score, for the trait domains of negative affectivity, antagonism, and disinhibition, and for the trait facets of emotional lability, manipulativeness, deceitfulness, and impulsivity. A small effect size was found for manipulativeness; however, medium effect sizes were obtained for the other traits in which significant differences between the groups were found.

Discriminant factor analysis results were in line with the aforementioned group differences. The best discriminators among patients with personality disorders vs. patients with other diagnoses included the domains of disinhibition, negative affectivity, and antagonism and the facets of impulsivity, deceitfulness, emotional lability, and manipulativeness. The PID5BF+M total score was also one of the best discriminators between the groups (fourth in the domain-level analysis and fifth in the facet-level analysis) confirming the potential utility of this indicator for detecting significant levels of personality dysfunction.

Finally, bearing in mind that the literature on the PID-5 suggests that ratings of 2 (sometimes true) or 3 (very true) have clinical relevance (23), our results indicate that individuals with rates above 2 in three or more of the 18 PID5BF+M facets may have a PD diagnosis. Considering the seven traits that significantly differentiate the groups (negative affectivity, antagonism, disinhibition, emotional lability, manipulativeness, deceitfulness, and impulsivity), two of these traits with rates above 2 are sufficient to distinguish the PD group from the non-PD group.

The prominence of maladaptive traits, particularly the presence of elevated scores in three or more facets in the PD group, mirroring the severity of personality dysfunction (6, 8, 20, 21), appears to support the ability of the PID5BF+M to differentiate PD from non-PD patients. Moreover, considering that the PD group was largely composed of borderline PD, characterized by the presence of facets from the negative affectivity, antagonism, and disinhibition domains in the AMPD, the results of the current study suggest that the PID5BF+M also has the potential to describe the specific traits that characterize the stylistic manifestations of PD. Therefore, although more research is needed, the current study appears to support the validity of the PID5BF+M as a global measure of personality dysfunction severity beyond just characterizing specific PD trait expressions.

The absence of other instruments' data to establish the convergent validity of the PID5BF+M, the heterogeneity of the clinical samples' composition and the small number of participants are limitations of this study. In particular, the fact that the PD sample has other diagnoses in comorbidity may overshadow the differences found. However, the study supports usage of the PID5BF+M for PD assessment, stressing its potential to identify patients with more severe personality dysfunction, that is, those who have higher scores in most of the maladaptive traits, and also highlighting the specific stylistic manifestations of the personality dysfunction. Moreover, considering that the PID5BF+M traits encompass traits described in both the ICD-11 PD classification system and the DSM-5 AMPD, it is plausible to expect that the current study, along with previous studies (14, 22), may contribute to future developments of the DSM-5, namely by bringing it closer to the ICD-11, the WHO authoritative mental disorders classification system. Finally, for clinical practice and the diagnostic process, it would be incommensurably fruitful to have a diagnostic tool with fewer items, which is less time consuming and bridges the ICD-11 and the DSM-5 personality disorders classification systems.

## Data Availability Statement

The data analyzed in this study is subject to the following licenses/restrictions: Our PID5BF+M data was derived from complete PID-5 data. To obtain the dataset analyzed for this study, please contact ruteoliveirapires@gmail.com or rpires@psicologia.ulisboa.pt.

## Ethics Statement

The studies involving human participants were reviewed and approved by Ethics committee of the Faculdade de Psicologia—Universidade de Lisboa. The patients/participants provided their written informed consent to participate in this study.

## Author Contributions

RP designed the study, provided state of the art revision, supervised the research project and data collection, performed the data analysis and its interpretation, and wrote the first draft of the manuscript. JH-C collaborated in the study design, supervised the research project and data collection, and provided critical revisions. AS performed and supervised the data analysis and interpretation. BB designed the study, provided state of the art revision, and provided critical revisions. MP, JG, AR, and JG supervised the data collection and provided critical revisions. BG collaborated in the study design, coordinated the research project and data collection, and provided critical revisions. All authors contributed to and have approved the final manuscript.

## Conflict of Interest

The authors declare that the research was conducted in the absence of any commercial or financial relationships that could be construed as a potential conflict of interest.
